# Hypermethylated in cancer 1(HIC1) suppresses non-small cell lung cancer progression by targeting interleukin-6/Stat3 pathway

**DOI:** 10.18632/oncotarget.8734

**Published:** 2016-04-14

**Authors:** Xiumin Wang, Yingying Wang, Gang Xiao, Jinglong Wang, Lidong Zu, Mingang Hao, Xueqing Sun, Yujie Fu, Guohong Hu, Jianhua Wang

**Affiliations:** ^1^ Department of Biochemistry and Molecular Cell Biology, Shanghai Key Laboratory of Tumor Microenvironment and Inflammation, Shanghai Jiao Tong University School of Medicine, Shanghai, China; ^2^ Cancer institute, Fudan University Shanghai Cancer Center, Shanghai, China; ^3^ Department of Chest Surgery, Renji Hospital, Shanghai Jiao Tong University School of Medicine, Shanghai, China; ^4^ Institute of Health Sciences, Shanghai Institutes for Biological Sciences, Chinese Academy of Sciences, Shanghai, China

**Keywords:** HIC1/IL-6 axis, hypermethylation, NSCLC

## Abstract

Non-small cell lung cancer (NSCLC), which accounts for more than 80% of lung cancers, is a leading cause of cancer mortality worldwide. However, the mechanism underlying its progression remains unclear. Here we found that HIC1 promoter was heavily methylated in NSCLC cell lines and tissues contributing to its low expression compared to normal controls. Restoring HIC1 expression inhibited migration, invasion and promoted inducible apoptosis of NSCLC cells. Notably, HIC1 is a tumor suppressor through inhibiting the transcription of IL-6 by sequence-specific binding on its promoter. Restoring IL-6 expression could partially rescue these phenotypes induced by HIC1 *in vitro and in vivo*. Mechanistic analyses show that autocrine secretion of IL-6 induced by loss of HIC1 activated STAT3 through IL-6/JAK pathway and was associated with NSCLC progression. The HIC1/IL-6 axis may serve as a prognostic biomarker and provide an attractive therapeutic target for NSCLC.

## INTRODUCTION

Lung cancer is the leading cause of cancer mortality worldwide, accounting for 1.6 million deaths according to the latest statistics [1]. Lung tumors are divided into two histological types: non-small cell lung cancer (NSCLC), which accounts for more than 80%, and small cell lung cancer (SCLC). Despite therapeutic advances, the overall 5-year survival of lung cancer remains about 17% [2]. Distant metastases in patients with lung cancer are the leading cause of death [3]. Therefore, it is significant to identify novel metastases-related genes in lung cancer, especially in NSCLC, which may become useful biomarkers for the early detection or attractive targets for treatment.

Recently, the importance of epigenetic changes that occur during lung cancer development has also been reported [4–7], prompting novel biomarkers for lung cancer. Hypermethylated in cancer 1(HIC1) is a gene located at 17p13.3 and can be activated by p53, which was first identified by Baylin in 1995 [8]. This finding also indicates that HIC1 expression is absent or decreased in neoplastic cells, which have the aberrant pattern of HIC1 CpG island *Not*I site methylation. Additionally, increasing evidence shows that HIC1 is aberrantly hypermethylated in multiple common types of human cancer tissues, including breast [9, 10], medulloblastomas [11, 12], gastric [13], hepatocellular carcinoma [14], colorectal [15], cervical [16] and lung tumors [17].

HIC1 is a sequence-specific transcriptional repressor that contains several autonomous repression domains [18–20], belonging to the BTB/POZ and C2H2 zinc fingers family [21]. The N-terminal BTB/POZ domain of HIC1 is involved in dimerization and in protein-protein interactions and the C-terminal region interacts with a specific DNA sequence, 5-^C^/_G_NG^C^/_G_GGGCA^C^/_A_CC-3 with a core GGCA motif [22]. *In vitro*, HIC1 is mainly a sequence-specific transcriptional repressor interacting with a still growing range of HDAC-dependent and HDAC-independent corepressor complexes [20]. Furthermore, it has been reported that the mice with homozygous disruption of HIC1 die perinatally and exhibit varying combinations of gross developmental defects [23]. In addition, the findings indicate that HIC1 inactivation may function as an initiating event in tumorigenesis based on the propensity of *Hic1^+/−^* mice to form spontaneous tumor [24] and the presence of HIC1 silencing events in pre-neoplastic conditions such as smoker's lung, colonic polyps and chronic hepatitis or cirrhosis [14, 15, 17, 25]. To date, HIC1 mutation in cancers has not been reported, therefore loss of its function mediated by epigenetic modification may drive key stages of human tumorigenesis. However, the role and mechanism of epigenetic silencing of HIC1 involved in the progression of NSCLC are still unknown.

Here, we investigated the methylation status of HIC1 promoter and the role of HIC1 plays in NSCLC. Our results indicate that IL-6, a critical cytokine for immune responses [26] and tumorigenesis [27], is a potential downstream target gene of HIC1. The alteration in the HIC1/IL-6 axis contributes to NSCLC progression and represents therapeutic targets.

## RESULTS

### Methylation of HIC1 promoter impairs its expression in NSCLC

**Figure 1:**
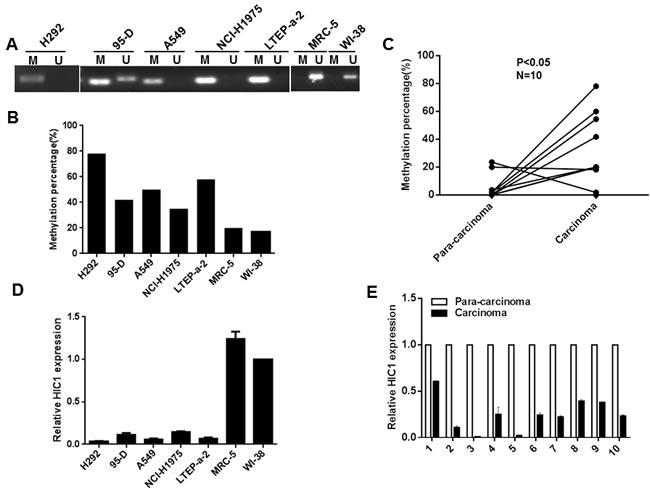
Methylation of HIC1 promoter impairs its expression in NSCLC **A.** Genomic DNAs from NSCLC cell lines were treated with sodium bisulfate, the PCR products amplificated with HIC1 MSP primers were confirmed by agarose gel electrophoresis. M: methylation; U: unmethylation. **B.** and **C.** Genomic DNAs from NSCLC cell lines and tissues were treated with sodium bisulfate, PCR products amplificated with HIC1 BSP primers were sequenced and the percentage of methylation was calculated. **D.** and **E.** Quantitative real-time RCR analysis of HIC1 gene in NSCLC cell lines and tissues.

Previous reports have indicated that HIC1 gene is silenced by DNA hypermethylation in various solid tumors [9, 14, 28, 29]. To examine whether HIC1 is inactive by hypermethylation in NSCLC, we examined the methylation status of HIC1 promoter in cell lines and 10 pairs of NSCLC carcinoma and para-carcinoma tissues by methylation specific PCR (MSP) and bisulfite sequencing PCR (BSP) (Figure 1A, 1B and 1C). Para-carcinoma tissues are more than 5cm away from the foci organization with the appearance of a normal non-cancerous infiltration. As shown in Figure 1A, one core promoter region was markedly methylated in H292, 95-D, A549, NCI-H1975 and LTEP-a-2 cells compared with normal human fetal lung fibroblast cells MRC-5 and WI-38 by MSP analyses. Next, the methylation percentage of 11 CpG sites located in −624 to −495bp upstream of the HIC1 transcription start site by BSP was further assayed. The results show that the methylation percentage of 11 CpG sites was greatly higher in H292, 95-D, A549, NCI-H1975, LTEP-a-2 cells than in MRC-5 and WI-38 cells (Figure 1B and Supplementary Figure 1A). Moreover, the percentage of methylated HIC1 promoter in 10 primary NSCLC carcinoma tissues was higher than in the respective para-carcinoma tissues (Figure 1C and Supplementary Figure 1B). We next explored the mRNA levels of HIC1 in cells and tissues by quantitative real-time PCR assays. The results show that HIC1 expression was lower in H292, 95-D, A549, NCI-H1975 and LTEP-a-2 cells (Figure 1D) and carcinoma tissues (Figure 1E) than in MRC-5, WI-38 cells and para-carcinoma tissues respectively. To explore whether regulating promoter methylation of HIC1 may affect its expression, we treated A549 and H292 cells with 5′;-Aza-CdR for 48 h. Quantitative real-time PCR and Western blot assays note that both mRNA and protein expression of HIC1 were somehow restored (Supplementary Figure 2A), accompanied by the attenuation of promoter methylation (Supplementary Figure 2B). Finally, immunohistochemical analyses of NSCLC tissue microarrays (TMAs) show that expression of nuclear HIC1 in para-carcinoma was 52.2%, while its expression in carcinoma was only 15.4% (Supplementary Figure 3). In addition, we found that nuclear HIC1 expression was correlated significantly with poorer pathological grading (*p* = 0.011). In total, these findings suggest that hypermethylation of the HIC1 promoter results in its impaired expression in NSCLC.

### HIC1 inhibits invasion, migration and promotes apoptosis of NSCLC cells

**Figure 2:**
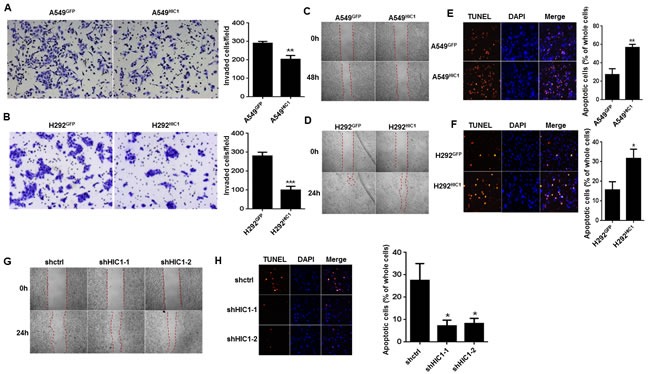
HIC1 inhibits invasion, migration and promotes apoptosis of NSCLC cells **A.** and **B.** The invasive properties of A549 and H292 cells expressing control vector or HIC1 were detected through extracellular matrices in porous culture chambers, the invasive cells were stained by crystal violet and then photographed by fluorescence inversion microscope system. **C.** and **D.** The migration abilities of A549 and H292 expressing control vector or HIC1 were detected by scratch wound healing assay. **E.** and **F.** A549 and H292 cells expressing control vector or HIC1 were treated with 0.1 μmol/L of staurosporine for 12 h, and apoptosis was performed by tunel assay using the In Situ Cell Death Detection Kit, TMR red (Roche). **G.** HIC1 knockdown by shHIC1-1 and shHIC1-2 in A549 cells promoted migration, **H.** inhibited apoptosis. The experiments were performed 3 times; representative images were shown (× 100). Data are represented as mean ± SD. *p < 0.05, **p < 0.01, ***p < 0.001.

Due to promoter hypermethylation, the silence of HIC1 is implicated in many canonical processes of cancer such as cell survival upon genotoxic stress [30], cell migration and motility [31]. To further explore the role of HIC1 in NSCLC, we restored HIC1 expression in A549 and H292 cells (noted as H292^HIC1^and A549^HIC1^) using lentivirus vector. The invasive capacity was significantly reduced in A549^HIC1^and H292^HIC1^cells compared with the respective controls using matrigel invasion assays (290 ± 10 *vs*. 203.3 ± 20.82; 280 ± 20 *vs*. 100 ± 20) (Figure 2A, 2B). Similarly, the ability of migration in A549^HIC1^ and H292^HIC1^ cells was also markedly inhibited using wound healing assays (Figure 2C, 2D). Apoptosis was induced through treating these cells with 0.1 μM staurosporine for 12 h [32]. TUNEL assay indicate that significantly increased number of apoptotic cells was observed in A549^HIC1^ and H292^HIC1^ cells as compared with controls (Figure 2E, 2F), which was further confirmed by quantitative analyses (27.33% ± 6.43% *vs*. 56.67% ± 3.51%; 15.67% ± 4.16% *vs*. 31.67% ± 4.73%). In contrast, compared with the control, shRNAs-mediated silence of HIC1expression, markedly promoted migration (Figure 2G) and decreased the number of apoptotic cells (27.5% ± 7.5% *vs* 7.23% ± 2.54%, 8.23% ± 2.36%) (Figure 2H). The results indicate that HIC1 may play a tumor suppressor role in NSCLC progression.

### HIC1 suppresses the expression of IL-6

**Figure 3:**
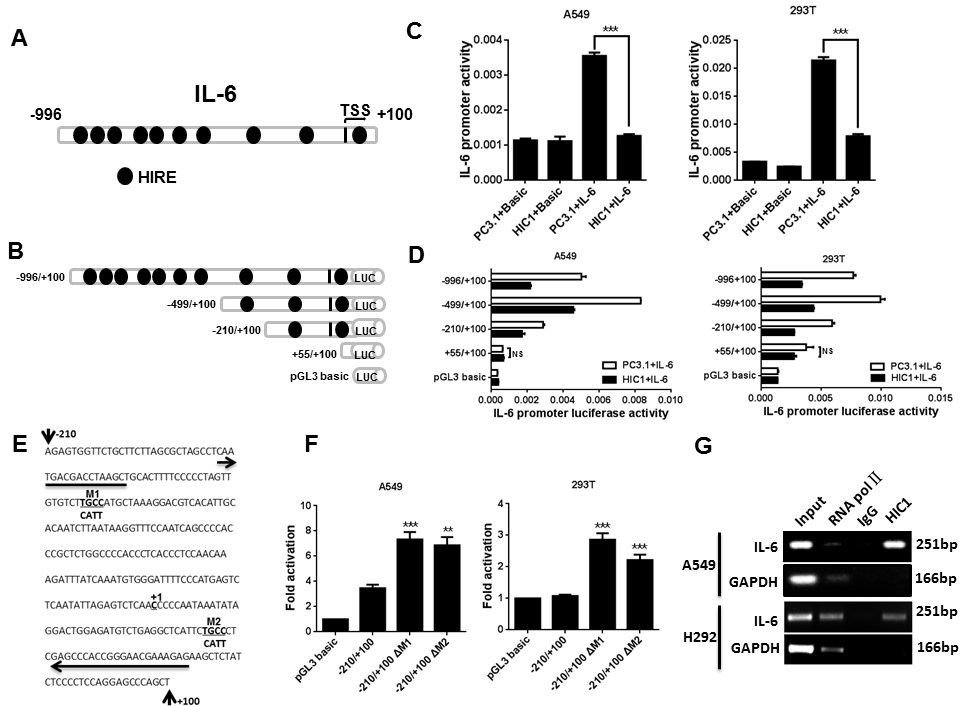
IL-6 is a direct target gene of HIC1 **A.** The map of IL-6 gene promoter. HIRE, HIC1 responsive element. **B.** The truncated IL-6 promoter was constructed to the pGL3-basic vector. The length of different promoter constructs used in reporter assays was shown. **C.** IL-6 promoter activity was detected in A549 and 293T cells co-transfected with full length construct (−996/+100) and PC3.1 or HIC1 expression vectors by luciferase reporter assay. Basic, control for promoter construct; PC3.1, control for HIC1 expression vector. **D.** The activities of IL-6 promoter were assayed in A549 and 293T cells co-transfected with truncated IL-6 promoter constructs and PC3.1 or HIC1 expression vector. NS, not significant. **E.** Nucleotide sequence of −210/+100 construct showed two potential HIC1 binding sites (TGCC). TGCC in the two sites was replaced by CATT and marked as M1 and M2. The arrows indicate the primers which are used to amplify the IL-6 promoter fragment in the ChIP experiment presented in Figure 3G. **F.** Luciferase reporter assays showed that ΔM1 and ΔM2 mutated construct significantly decreased the repression ability of HIC1. **G.** ChIP analysis of HIC1 binding to the IL-6 promoter region in A549 and H292 cells. Three independent experiments were performed. Data are represented as mean ± SD. **p < 0.01, ***p < 0.001.

To explore potential downstream targets of HIC1 involved in above effects, we analyzed our previous genome-wide transcriptome profile of MDA-231^HIC1^ *vs*. MDA-231^GFP^ cells and C4-2B^HIC1^ *vs*. C4-2B^GFP^ cells by Agilent Whole Human Genome Microarrays [33, 34] (Supplementary Figure 4A). Given the pro-migration [35] and anti-apoptosis roles [36] of IL-6 in NSCLC reported by previous researches, among the genes markedly regulated in these cells, we focused on IL-6. Also, IL-6 levels are significantly elevated in lung cancer patients, associated with poor prognosis [37, 38]. Additionally, 10 putative HIC1-responsive elements (HiRE, GGCA or TGCC) [22] are noted in the regulatory region of IL-6 promoter (Figure 3A). Based on these findings, we next explored the mechanism of how HIC1 modulates IL-6 expression in NSCLC cells.

When restoring HIC1 expression in A549 cells and H292 cells, detected by real-time PCR (Supplementary Figure 4B, 4C), IL-6 transcription was reduced obviously in both cells (Supplementary Figure 4B, 4C). In addition, ELISA assays show that IL-6 secretion was also decreased (Supplementary Figure 4D, 4E). In contrast, HIC1 knockdown by shRNAs in A549 cells markedly enhanced IL-6 levels (Supplementary Figure 4F, 4G). These findings suggest that expression of IL-6 could be inhibited by HIC1.

We next investigated whether HIC1 could inhibit the activity of IL-6 promoter, the −996/+100 promoter region of IL-6 was cloned into the pGL3 - Basic reporter vector, and a series of IL-6 truncated promoter/reporter fusion plasmids containing progressive 5′;deletions from −996 to +100 were constructed with gradually eliminated the putative HiREs (Figure 3A, 3B). These constructs were then transfected alone or with the pcDNA3.1-HIC1 expression vector into A549 and 293T cells, and promoter activities were thus measured by luciferase reporter assays. Figure 3C shows that the activity of IL-6 promoter was more than 3-fold higher than the basic group. However, transient transfection of HIC1 significantly inhibited the IL-6 promoter activity in both cells (Figure 3C).

Notably, the capacity of HIC1 to repress IL-6 promoter activity in A549 and 293T cells was still markedly remained when co-transfected with −996/+100, −499/+100 or −210/+100 truncated constructs, but the effect was almost lost with +55/+100 truncated construct which doesn't contain HIC1 binding sites (Figure 3D). These results suggest that the regulatory region involved in the HIC1-mediated repression of IL-6 may be located in the −210 bp to +55 bp region of the promoter which contains two putative HIC1 binding sites, M1, M2 (Figure 3E). We thus mutated this two HiRE sites (TGCC into CATT) to abolish HIC1 binding, respectively (Figure 3E). Figure 3F shows that both mutated constructs significantly decreased the repression capacity of HIC1 in two cell lines as compared with the controls. Thus, these results demonstrate that the two putative M1, M2 sites in IL-6 promoter are both essential for HIC1-mediated repression. Finally, to further confirm whether HIC1 could bind on IL-6 promoter, ChIP assays were performed in A549 and H292 cells using HIC1 polyclonal antibody and the pull-down DNA was then amplified by PCR assays. The primers were designed on the −181bp to +70bp of IL-6 promoter to amplify the region mediating the repressive effects of HIC1. Figure 3G shows that the indicated IL-6 promoter region was markedly amplified from the HIC1-immunoprecipitated A549 and H292 chromatins, but absence from chromatin immunoprecipitated by the control rabbit IgG.

Taken together, these findings confirm that the binding of HIC1 proteins on IL-6 promoter enables it to repress IL-6 expression, while reducing HIC1 expression by hypermethylation in NSCLC is likely to increase the expression of IL-6, associated with NSCLC progression.

### HIC1 inactivates the activity of STAT3 through targeting IL-6/JAK pathway

Based on these results, we next assayed which pathway may be involved in HIC1-inhibited IL-6 expression in NSCLC progression.

Firstly, we examined the levels of p-STAT3^Y705^, p-ERK1/2^T202/Y204^ and p-NF-κB p65^S536^ in A549 and H292 cells restoring HIC1 expression by Western blot analyses. The results show that p-STAT3^Y705^ level was markedly reduced in both cells compared with the respective controls (Supplementary Figure 5A), but no effect was observed on the ERK1/2 and NF-kB p65 pathways. In contrast, HIC1 knockdown by shRNA1 and shRNA2 could enhance the phosphorylation and activity of STAT3 (Supplementary Figure 5B, 5C).

**Figure 4:**
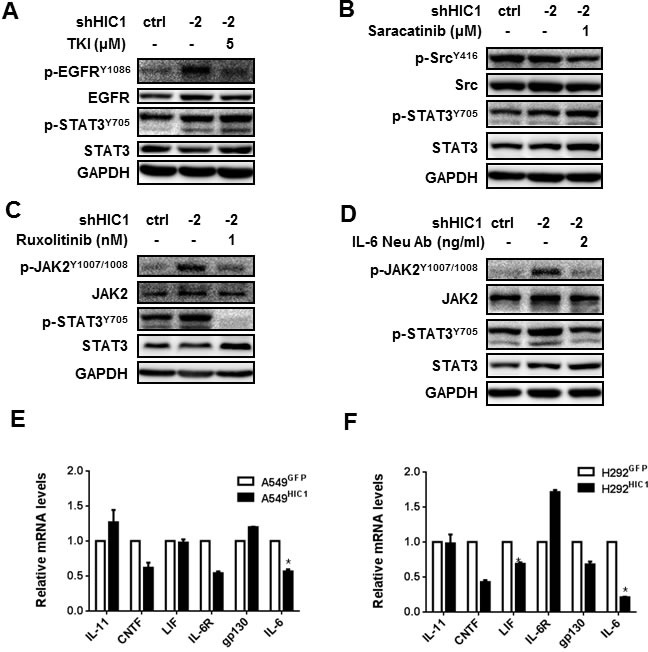
HIC1 inactivates the activity of STAT3 through targeting IL-6/JAK pathway A549 cells with HIC1 knockdown were treated with multiple signaling pathways inhibitors for 6 hours. The activities of these pathways were detected by Western blot, **A.** 5μM TKI reduced the levels of p-EGFR^Y1086^, but had no effect on p-STAT3^Y705^. **B.** 1μM Saracatinib reduced the levels of p-SRC^Y416^, but had no effect on p-STAT3^Y705^. **C.** and **D.** 1 nM Ruxolitinib and 2 ng/ml IL-6 Neu Ab reduced the levels of both p-JAK2^Y1007/1008^ and p-STAT3^Y705^. **E.** and **F.** Quantitative real-time PCR assayed the expression of IL-6 family factors such as IL-11, CNTF, LIF, IL-6 and the receptors IL-6R and gp130 in A549 ^HIC1^ and H292^HIC1^ cells and their controls. Three independent experiments were performed. Data are represented as mean ± SD.*p < 0.05.

Next, we assayed the potential mechanism of how HIC1 affects p-STAT3^Y705^ level. A549 cells with HIC1 knockdown were treated with TKI or Saracatinib, the inhibitor for EGFR or Src pathway, respectively. Figure 4A and 4B indicate that these treatments almost had no effect on the phosphorylation of STAT3^Y705^. However, the p-STAT3^Y705^ level could be greatly reduced by JAK pathway inhibitor Ruxolitinib or IL-6 neutralizing antibody (Figure 4C, 4D), which suggests that HIC1 reduces the activity of STAT3 through inhibiting IL-6/JAK pathway.

Finally, we assayed whether HIC1 could repress other IL-6 family factors such as IL-11, LIF and CNTF or the receptors IL-6R and gp130 in A549 and H292 cells. As shown in Figure 4E and 4F, IL-6 was the only significantly reduced gene in both HIC1 restored expression cells compared with the respective control, yet other factors or receptors were not consistently inhibited. Therefore, these findings demonstrate that HIC1 suppresses the activity of STAT3 through inhibiting IL-6/JAK pathway.

### IL-6 partially rescues HIC1-induced phenotypes of NSCLC cells

**Figure 5:**
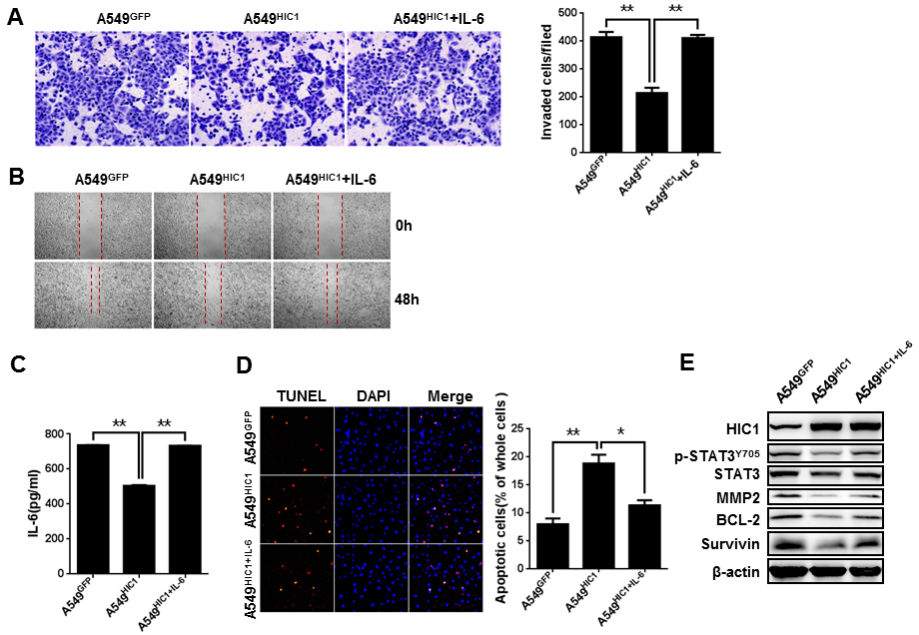
IL-6 partially rescues HIC1-induced phenotypes of NSCLC cells **A.** and **B.** Exogenous IL-6(40 ng/ml) stimulation in A549 cells could partially rescued the reducing invasion and migration caused by HIC1 re-expression. **C.** ELISAs determined the expression of IL-6 in A549^GFP^, A549^HIC1^and A549^HIC1+IL-6^ cells. **D.** Re-expression of IL-6 in A549^HIC1^ cells partially rescued HIC1-induced enhancement of apoptosis. Representative images were shown (× 100). **E.** p-STAT3^Y705^, MMP2, Bcl-2 and Survivin levels were detected in A549^GFP^, A549^HIC1^ and A549^HIC1+IL-6^ cells by Western blot. Three independent experiments were performed. Data are represented as mean ± SD. *p < 0.05, **p < 0.01.

To further confirm that IL-6 is a critical target gene in HIC1-induced phenotypes, we firstly assayed the effect of IL-6 on cell invasion and migration *in vitro*. The results indicate that exogenous IL-6 stimulation on A549^HIC1^ cells could partially rescued the reduced invasion and migration caused by HIC1 re-expression (Figure 5A, 5B). Similar effect was observed in H292^HIC1^ cells (Supplementary Figure 6A, 6B). Meanwhile, using lentiviral infection, IL-6 expression was restored in A549^HIC1^ and H292^HIC1^ cells, as confirmed by ELISAs (Figure 5C and Supplementary Figure 6C). Figure 5D and Supplementary Figure 6D show that re-expression of IL-6 in A549^HIC1^ and H292^HIC1^ cells partially rescued HIC1-induced enhancement of apoptosis compared with the respective controls. In addition, to further explore whether HIC1 exerts these effects through targeting IL-6/JAK/STAT3 pathway, we examined its downstream targets, including MMP2, Bcl-2 and Survivin. As expected, re-expression of IL-6 in A549^HIC1^ and H292^HIC1^ cells somehow rescued HIC1-induced reduction of p-STAT3^Y705^, Bcl-2, Survivin and MMP2 (Figure 5E and Supplementary Figure 6E).

In contrast, knockdown IL-6 by shRNA in A549 cells in which HIC1 has already been knockdown (noted as A549^shHIC1-1/2+shIL-6^), as confirmed by Western blot and ELISAs (Supplementary Figure 7A, 7B), we found that the reduced apoptosis was greatly enhanced compared with the A549^shHIC1-1/2^ cells (Supplementary Figure 7C). Similarly, the effect was observed in A549^shHIC1-1/2^ cells treated with STAT3 inhibitor S3I-201(Supplementary Figure 7D). Meanwhile, the phosphorylation (Supplementary Figure 7E) and the activity of STAT3 (Supplementray Figure 7F) were both markedly reduced in A549^shHIC1-1/2+shIL-6^ cells compared with the A549^shHIC1-1/2^ cells.

### Restoring HIC1 expression inhibits tumor metastasis *in vivo*

**Figure 6:**
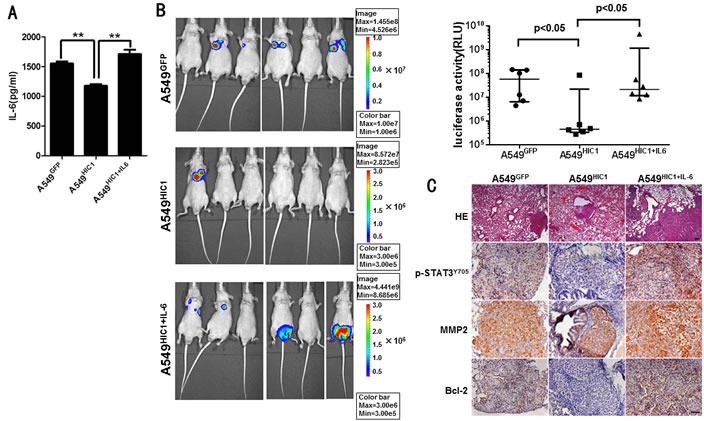
Effect of restoring HIC1 expression on tumor metastasis *in vivo* **A.** ELISA assays detected the level of IL-6 secretion in luciferase-tagged A549^GFP^, A549^HIC1^ and A549^HIC1+IL-6^ cells. **B.** Restoring HIC1 expression in A549 cells significantly reduced lung metastasis compared with the control group (*p* < 0.05) in transplantable nude balb/c mouse models, but re-expression of IL-6 in A549^HIC1^ cells(noted as A549^HIC1+IL-6^) partially rescued HIC1-induced reduction of metastasis compared with the A549^HIC1^ cells (*p* < 0.05). On the left, representative images of BLI animals at the eighth week were shown. The up right statistical graph showed the fluorescence signal intensity collected from the metastasis loci of each mouse. Each of group *n* = 6, the *p* value was calculated by Mann Whitney U test. **C.** HE staining of tumor tissue and immunohistochemical evaluation of p-STAT3^Y705^, MMP2 and Bcl-2 in lung micrometastases grown in nude *balb/c* mice. Representative microscopic images of HE staining (×50, scale bar, 100μm) and tumor tissues stained with an anti-human p-STAT3^Y705^ antibody (1:50), MMP2 antibody (1:100) and Bcl-2 antibody (1:100) (× 200, scale bar, 50μm) were shown.

Finally, to further explore whether HIC1 is involved in inhibiting tumor metastasis *in vivo*, we transplanted these luciferase-tagged A549 cells into nude balb/c mice by tail vein injection. IL-6 secretion from the luciferase-tagged A549 cells before and after rescuing its expression was detected by ELISA assays (Figure 6A). The results show that HIC1 significantly reduced lung metastasis in 8^th^ week compared with the control group. However, re-expression of IL-6 in A549^HIC1^ cells partially rescued HIC1-induced reduction of metastasis (Figure 6B). Immunohistochemistry staining for the mice lung tissues show that the expression of p-STAT3^Y705^, MMP2 and Bcl-2 were repressed in the micrometastases produced by A549^HIC1^ cells, whereas the effects could be restored in A549^HIC1+IL-6^ cells (Figure 6C). These results suggest that HIC1 can significantly inhibit metastasis and promote apoptosis of NSCLC cells *in vivo* by inactivating STAT3 pathway.

### HIC1/IL-6 axis predicts clinical outcome in NSCLC patients

Based on these findings *in vitro* and *in vivo*, we assess whether HIC1/IL-6 axis is responsible for clinical outcome in NSCLC patients. Using clinical microarray database of NSCLC [39], we found that significantly poorer overall survival (OS) and metastasis free survival (MFS) were observed in NSCLC patients with low HIC1 and high IL-6 expression than the patients with high HIC1 and low IL-6 expression(Supplementary Figure 8A, 8B). The finding suggests that the expression of HIC1 may be negatively correlated with IL-6 in the progression of NSCLC, which was further confirmed by tissue microarray staining in NSCLC clinical samples (Supplementary Figure 9).

**Figure 7:**
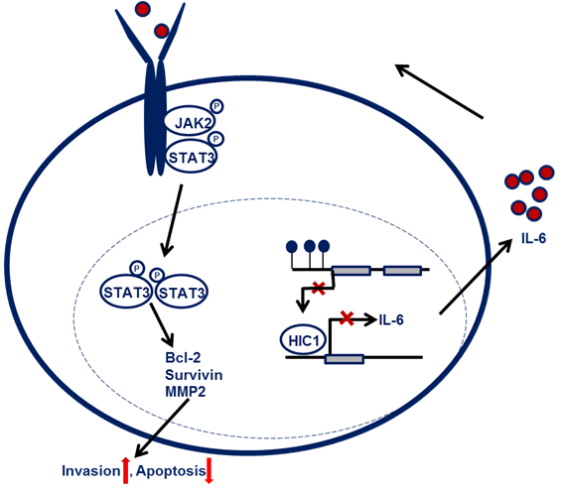
Schematic model for the function of HIC1/IL-6 axis in NSCLC progression HIC1 expression is impaired in NSCLC due to the hypermethylation modification, leading to the higher secretion of IL-6. Autocrine of IL-6 can activate JAK/STAT3 pathway and the expression of downstream targets, such as MMP2, Bcl-2 and Survivin, therefore promoting NSCLC progression.

Taken together, schematic model is showed for the role of HIC1/IL-6 axis in NSCLC progression. HIC1 expression is impaired in NSCLC due to the hypermethylation modification, leading to the higher secretion of IL-6. Autocrine of IL-6 can activate JAK/STAT3 pathway and the expression of downstream targets, such as MMP2, Bcl-2 and Survivin, therefore promoting NSCLC progression (Figure 7).

## DISCUSSION

Recently, our and other studies have indicated that HIC1 is frequently hypermethylated in a variety of solid tumors and leukemia [12, 14, 15, 34, 40, 41], However, the consequences of epigenetic modification on HIC1 promoter in NSCLC remain unclear. In this investigation, our data show that HIC1 promoter hypermethylation exists in NSCLC (Figure 1, Supplementary Figure 1 and Supplementary Figure 10), which results in its low-expression and is potentially responsible for NSCLC progression.

Indeed, our studies indicate that restoring HIC1 expression markedly inhibited migration, invasion and promoted apoptosis in NSCLC cells, whereas reduction of HIC1 expression by shRNAs led to the opposite effects. In addition, *in vivo* experiments show that restoring HIC1 expression had a marked effect on reducing lung metastases. Analyzing our previous microarray data and the subsequent *in vitro* experiments, we identified IL-6 as the downstream target which could partially rescued HIC1-induced phenotypes.

IL-6 is a critical cytokine for immune responses [26] and tumorigenesis [27]. Early studies implicated IL-6 and its major effector STAT3 as pro-tumorigenic agents in many cancers, including breast, lung, colon, prostate, ovarian, and hematological cancers as well as melanoma [42]. IL-6 is significantly elevated in lung and breast cancer patients, associated with poor prognosis [37, 38, 43]. Notably, IL-6 has been reported to correlate with drug resistance [44–46]. Therefore it is critical to identify upstream regulator of IL-6, which may provide some useful clues for NSCLC progression and treatment.

In this study, we further confirm that endogenous HIC1 expression could bind to the promoters of IL-6, and inhibit its expression. It is well known that IL-6 signals *via* a heterodimeric IL-6R/gp130 complex, whose engagement triggers activation of Janus (JAK) kinases, and the downstream effectors STAT3, involved in cell apoptosis and invasion/migration through activating some critical targets including Bcl-2, Survivin, and MMP2 [47]. Previous reports have shown that activation of STAT3 can be also triggered by EGFR, Src [48] or HGF/MET pathway [49]. However, we here found that HIC1 reduces the activity of STAT3 only through inhibiting IL-6/JAK pathway, but not through EGFR or Src pathway. In addition, we found the expression of HGF and its receptor MET could not be inhibited by HIC1 through analyzing our previous genome-wide transcriptome profile. So we speculated that STAT3 may also not be activated in response to HGF activation of its receptor MET. Furthermore, restoring expression of HIC1 in cells only significantly reduced IL-6 expression, but not other factors or receptors. Given the fact that IL-6R is a STAT3 transcription target [50], the difference in IL-6R mRNA levels in A549^HIC1^ *versus* A549^GFP^ compared to those in H292^HIC1^ *versus* H292^GFP^ may result from other mechanisms in H292 cells which could also regulate IL-6R, for example, the sex-based difference between A549 and H292 cell line origin maybe a possible contribution [51]. Notably, the mechanism underlying IL-6 expression inhibited by HIC1 has not been previously reported to the best of our knowledge.

Previous reports have showed that low HIC1 expression is involved in the malignant progression of NSCLC [52, 53]. Here, using the clinical microarray database of NSCLC [39], we disclosed that NSCLC patients with low level of HIC1 and high IL-6 expression had greatly poorer OS and MFS than the patients with high level of HIC1 and low IL-6 expression. The findings suggest that the expression of HIC1 is negatively correlated with IL-6 level in the progression of NSCLC patients.

In summary, our results suggest that HIC1/IL-6 axis may serve as a prognostic factor of NSCLC progression and provide an attractive therapeutic target.

## MATERIALS AND METHODS

Some reagents, antibodies, and plasmids are listed in Supplementary Methods.

### Cell culture

Human non-small cell lung cancer cell lines A549, H292, NCI-H1975 (obtained from American Type Culture Collection) and 95-D, TLEP-a-2 (obtained from the Institute of Health Sciences, SIBS, CAS / SJTUSM) were cultured in RPMI-1640 (Hyclone) with 10% FBS (GIBCO), MRC-5, WI-38 (obtained from American Type Culture Collection) were cultured in Eagle's Minimum Essential Medium (GIBCO) with 10% FBS (GIBCO), luciferase-tagged A549 cells (purchased from Shanghai BioDiagnosis Co., Ltd) were cultured in Ham's F-12K (Kaighn's) Medium (GIBCO) with 10% FBS (Sigma). The cell lines were tested and authenticated by DNA typing in Shanghai JiaoTong University Analysis Core (last test in December 2014) and were cultured at 37°C water-saturated 5% CO_2_ atmosphere.

### NSCLC tissues profile

Carcinoma and corresponding para-carcinoma tissue samples of 10 NSCLC patients were obtained from Department of Thoracic Surgery, Renji Hospital, Shanghai Jiaotong University School of Medicine. All specimens were pathologically and clinically diagnosed as NSCLC, the lung carcinoma and para-carcionoma tissues (more than 5cm away from the cancer lesions with appearance of normal non-cancerous infiltration) through Surgical resection consisting of paired specimens. More details for the samples are shown in Supplementary Table 2. Patients’ consent and approval from the shanghai Jiao Tong University School of Medicine Ethics Committee were obtained before using these clinical materials for research purposes.

### Methylation analyses

Methylation analysis was performed according to procedures described previously [34]. Methylation-specific PCR(MSP) primers were designed according to the literature [54]: M sense primer : 5′;-GGTAGGGGAGTTTAGGGT TC-3′;and M antisense primer: 5′;-TTCCAACTA C A AACAAAACGAA-3′;, and U sense primer: 5′;-GGGTAGGGGAGTTTAGGGTTT-3′; and U antisense primer: 5′;-AATTTTCCA ACTACAAACAAAACAAA-3′;. CpG islands located in 1,000 bp upstream from the TSS of HIC1 gene subtype1 were identified using Methprimer software. The selected primers for bisulfate-sequencing PCR (BSP) as follows: sense primer: 5′;-GGGTAGGGGAGTTTAGGGTT-3′;and antisense primer : 5′;-AAAAAAATTTTCC AACTACAAACAAAA-3′;. Genomic DNAs from cell lines and tissues were treated with sodium bisulfate (ZYMO Research Corp, California, USA), and then analyzed by MSP or BSP. The PCR products were confirmed by agarose gel electrophoresis and visualized using ethidium bromide staining for MSP. Amplifiled BSP products were subcloned into the TA vector system (Invitrogen Corp, Carlsbad, CA) according to the manufacturer's instructions. DNA sequencing was performed on five to ten individual clones (Invitrogen Corp, Carlsbad, CA).

### Western blotting analyses

Cells were washed twice with PBS and directly lysed in RIPA buffer (Cat#: 89900, Thermo Scientific, Waltham, MA) containing a protease inhibitor mixture (KangChen Bio-tech, shanghai, China). Western blot was performed as previously described [33]. Results are representative of at least three experiments. Details are provided in the Supplementary methods section.

### RNA extraction and quantitative real-time PCR

Details are provided in the Supplementary methods section.

### Construction of stably overexpression or knockdown cells

For overexpression of HIC1 in NSCLC cells, human full-length HIC1 cDNA was inserted into lentivirus vector pHR-SIN-CSIGW. Lenti-x cells were then transfected with the PMD2.G, PSPAX2 and HIC1 expression vector using the lipofectamine 2000 (Invitrogen). After 48 h, culture supernatants were collected and passed through 0.45μm filters, mixed with fresh media (1:1) and polybrene (8 μg/ ml) to infect target cells. The cells which restored expression of HIC1 were noted as A549^HIC1^ and H292^HIC1^, and the respective control were noted as A549^GFP^ and H292^GFP^ cells. For generation of a stable HIC1 knockdown cell line, GV248 lentiviral vectors expressing short hairpin RNAs targeting HIC1 were purchased from GeneChem Company (Shanghai, China). Lentiviruses were produced as described above and infected cells were selected by puromycin (1 μg/ml) for 2 days. The seed shRNA sequences are as follows: shHIC1-1: TGTGCAAGAAACGCCTCAA; shHIC1-2: TGGCGCAGA CCA CGCACTT. For generation of a stable IL-6 knockdown cell line, p-LVX-shRNA2 vectors expressing short hairpin RNAs targeting IL-6 were constructed and the seed shRNA sequence: CTCAAATAAATGGCTAACTTA. Lentiviruses were produced as described above. Human full-length IL-6 cDNA was inserted into lentivirus vector pLenti-easy-HA, Lentiviruses were produced as described above and infected cells were selected by G418 (800 ng/ml) for 5 days.

### Luciferase reporter assays

IL-6 promoter regions at −996 / +100, −499 / +100, −210 / +100 and +55 / +100 were generated from genomic DNA of A549 cells. These promoter sequences were cloned into the pGL3-Basic reporter vectors and verified by sequencing. We made point mutations into pGL3-Basic-210/+100 constructs by using KOD-Plus Mutagenesis kit (Cat#: SMK-101, TOYOBO). Details are provided in the Supplementary methods section.

### Chromatin immunoprecipitation

Chromatin Immunoprecipitation (ChIP) was performed according to published protocols with slight modifications [55]. Details are provided in the Supplementary methods section.

### Migration and invasion assays

The migration status was assessed by measuring the movement of cells into a scraped area created by a 200 μl pipette tube. After scratched, cells were cultured in medium supplement with 1% FBS to eliminate the effect of cell proliferation. Cell invasion was examined using a reconstituted extracellular matrix membrane (BD Biosciences, San Jose, CA). Cells suspended at 5×10^4^ cells/0.5 ml in serum-free medium were placed in the top chambers, and complete medium containing 10% fetal bovine serum (FBS) was added to the bottom chambers. The chambers were then incubated for 24 h at 37°C with 5% CO_2_. After incubation, the medium were completely removed from the top and bottom wells, the chambers were fixed with methanol for 30 min and stained with crystal violet for another 30 min. The non-invasive cells were gently removed from the top wells with a cotton-tipped swab and cell counting was facilitated by photographing the membrane through the microscope (Zeiss) under ×10 objective lens.

### Immunohistochemistry

Tissue microarray and slides of lung tissues containing micrometastases produced from A549^GFP^, A549^HIC1^ and A549^HIC1+IL-6^cells in mice were deparaffiniced in Histoclear solution (National Diagnostics, Atlanta, GA) and rehydrated in a series of ethanol. Antigen was retrieved with the use of 10 mM sodium citrate buffer (pH 6.0) at 100°C for 10 min, and then treated with 0.3% hydrogen peroxide. After blocking with normal goat serum for 30 min, tissues were incubated with primary antibody at 4°C overnight. HIC1 (Sigma), IL-6 (Abcam), p-STAT3^Y705^ (Abcam), MMP2 (Santa Cruz) and Bcl-2 (CST) were used as 1:200, 1:200, 1:50, 1:100 and 1:100 dilutions respectively. Then tissues were incubated with appropriate biotinylated secondary anti-rabbit antibody (1:200, Vector Laboratories, Burlingame, USA) for 30 min, followed by incubation with the avidin-biotin-complex-PO using the VECTASTATIN^®^ Elite^®^ ABC kit (Vector Laboratories, California, USA) and developed in DAB Coloring Agent (Sigma, St Louis, MO, USA). we determined a general intensity score value of 0 to 3 and multiplied this value by the percentage of HIC1 - (nuclear) or IL-6 (cytoplasm) - positive tumor cells score value of 0 to 4 for a final HIC1 or IL-6 score of 0 to 12. For the analysis presented in this study, 0≤score<6 was defined as negative staining, and 6≤score≤12 was defined as positive group. The images were photographed by the microscope (Zeiss) under ×20 and ×40 objective lens.

### Apoptosis assays

Apoptosis assays were performed according to the manufacturer's protocol. The medium was removed from 24 - well plates and the slides were rinsed twice with 200 μl PBS, for 5 minutes each. Then, 50 μl Tunnel reaction mixture was added on cells which were adhered to the slides and the slides were incubated in a humidified atmosphere for 60 min at +37°C in the dark. After incubation, the slides were rinsed for 3 times with 200 μl PBS, 5 minutes each, then 200 μl 0.5 μg/ml DAPI solution were added into 24 - well plates and the slides were incubated for 5 min in the dark. Finally, the slides were embedded with antifade and were analyzed and photographed under a fluorescence microscope (Zeiss) with ×10 objective lens.

### Tumor metastasis assays *in vivo*

All experimental animal procedures were performed in compliance of the institutional ethical requirements and were approved by the Shanghai Jiao-Tong University School of Medicine Committee for the Use and Care of Animals. The modified transplantation through tail vein injection was performed as described previously [34]. Briefly, 5- to 6-week-old nude *balb/c* mice (Slaccas Laboratory Animal, Shanghai, China) were anesthetized, luciferase tagged A549^GFP^, A549^HIC1^ and A549^HIC1+IL-6^ cells (2 × 10^6^ / 150 μl / mouse) were respectively injected into tail vein using a 1 ml syringe fitted with a 25-gauge needle. After 8 weeks, bioluminescence was utilized to follow tissue metastases. The mice were injected intraperitoneally with 200 μl of 15 mg/ml luciferin prior to the Xenogen IVIS cryogenically cooled imaging system to detect the tumor metastatic sites.

### Tissue microarray and immunohistochemistry

High-density tissue microarray of NSCLC (Cat#:HLug-Ade150 Sur −02) was purchased from Outdo Biotech, details of the procedure are described in the immunohistochemistry section.

### ELISA

The secreted IL-6 in condition medium for cell cultures was detected by using Quantikine Human IL-6 ELISA Kits (Cat#:DY206, R&D systems). ELISA was done according to the manufacture's instruction. All experiments were done with 4 wells per experiment and repeated three times.

### Statistical analyses

HIC1 staining in tissue microarray was performed with Pearson's Chi-Square Test. The expression correlation between HIC1 and IL-6 was tested by Spearman's correlation analysis. Bioluminescent imaging (BLI) signal in live animals was calculated by Mann Whitney U test. For survival analyses, overall survival (OS) and metastasis free survival (MFS) stratified by expression of the gene of interest, were presented as Kaplan-Meier plots and tested for significance using log-rank tests. Other data were presented as mean SD and analyzed using Student's t test, 2-tailed. Values of *p* < 0.05 were considered statistically significant.

## SUPPLEMENTARY MATERIAL FIGURES AND TABLES


