# Supplementary material for: Hypermethylated in cancer 1(HIC1) suppresses non-small cell lung cancer progression by targeting interleukin-6/Stat3 pathway
Source: Oncotarget. 2016 Apr 14;7(21):30350–64. doi: 10.18632/oncotarget.8734 (PMC5058685; doi:10.18632/oncotarget.8734)
Supplement: Supplementary file 1 [file oncotarget-07-30350-s001.pdf]

Hypermethylated in cancer 1(HIC1) suppresses non-small cell lung cancer progression by targeting interleukin-6/Stat3 pathway

Supplementary Material

Western blotting antibodies and reagents

The commercial antibodies of the following were used: anti-human Phospho-STAT3Y705 (Cat#: 9145), STAT3 (Cat#: 4904), EGFR (Cat#: 4267), Phospho-SrcY416 (Cat#: 6943), Src (Cat#: 2108), Phospho-NF-κB p65S536 (Cat#: 3033), Phospho-ERK1/2T202/Y204 (Cat#: 4376), ERK1/2 (Cat#: 9102), NF-κB p65 (Cat#: 4764), caspase3 (Cat#: 9662), Cleaved caspase3 (Cat#: 9664), Bcl-2 (Cat#: 2870), Survivin (Cat#: 2808), were purchased from Cell Signaling Technology (CST, Boston, USA). Phospho-EGFRY1086 (Cat#: 2284-1), Phospho-JAK2Y1007/1008 (Cat#: 1477-1), JAK2 (Cat#: 2863-1) were purchased from Epitomics and diluted in 1:1000 in Western blot. Other commercial antibodies used were as follows: GAPDH (Cat#: KC-5G4) was purchased from Kang Chen Bio-tech (Shanghai, China), HIC1 (Cat#: H8539), β-Actin-dase (Cat#: A3854) were purchased from Sigma and diluted in 1:5000. MMP2 (Cat#: sc-10736) were purchased from Santa Cruz Biotechnology and diluted in 1:500 in assays. The EGFR inhibitor TKI (erlotinib)(Cat#: S7786), Src inhibitor Saracatinib(Cat#: S1006), JAK inhibitor Ruxolitinib (Cat#: S1378), apoptosis-inducing agents Staurosporine(Cat#: S1421) and STAT3 DNA-binding activity inhibitor S3I-201 (Cat#: S1155) were purchased from Selleckchem, IL-6 Neu Ab(IL-6 neutralization antibody) (Cat: #6708) was purchased frorm R&D systems. Recombinant Human IL-6(Cat:#200-06) was purchased from PeproTech.

RNA extraction and quantitative real-time PCR

Total RNA was extracted using the Trizol reagent (Cat#: 15596-026, Invitrogen) and reverse transcribed using the transcriptase cDNA synthesis kit (Fermentas) according to the manufacturer’s instructions. One microgram of total RNA was reverse

transcribed into cDNA and an equal volume of cDNA was used as the PCR template using specific primers forIL-6 (Genbank No: NM_000600.3) and HIC1 (Genbank No: NM_006497.3). Real-time PCR analysis was performed by SYBR Premix Ex TaqTM(Cat#: RR420A, TaKaRa, Dalian, China) in an Applied Biosystems 7500 Fast Real-Time PCR System (ABI, USA) according to the manufacturer's instructions. Primers were used at a concentration of 0.5 µM. According to the melting point analysis, only one PCR product was amplified under the following conditions: initial denaturation at 95°C for 30 sec, denaturation at 95°C for 5 sec, annealing at 60°C for 30 sec, for a total of 40 cycles. GAPDH is used as the reference gene to normalize the gene expressions. The primers are shown in Supplementary table 1.

Luciferase reporter assays

The HIC1 plasmid, IL6 promoter constructs and Renilla plasmid DNA (200ng: 200ng: 20ng) were co-transfected into A549 and 293T cells for 6 - 9 h and then incubated for 24 h in fresh complete medium. Cells were then rinsed in cold PBS and lysed with the luciferase assay buffer. Luciferase activities were measured by using a dual luciferase assay kit (Cat#: E1960, Promega, Wisconsin, USA) with a Berthold chemiluminometer (Berthold Detection Systems GmbH)[1]. The results were expressed as ratio of firefly luciferase activity to Renilla luciferase activity. Data were expressed as the mean values and standard deviations from at least three independent transfections. PSTAT3-TA-Luc (Cat:D2259) was obtained from Beyotime Institute of Biotechnology[2]. The pSTAT3-TA-Luc: Renilla plasmid DNA (400ng: 20ng) were co-transfected into the same concentration of A549-shHIC1-ctrl, A549-shHIC1-1, A549-shHIC1-2, A549-shHIC1-1+shIL6, A549-shHIC1-2+shIL6 cells in 24-well for 6 - 9 h and then incubated for 24 h in fresh complete medium. Luciferase activities were measured as above.

Chromatin Immunoprecipitation

Formaldehyde was added directly to the cultured A549 cells to a final concentration of 1% for 10 min at room temperature. The cross-linking was stopped by adding

glycine to a final concentration of 0.125 M. After incubating for 5 min, cells were scraped from dish into a conical tube. Then, the samples were pelleted, resuspended in nuclear lysis buffer, and sonicated to obtain chromatin fractions with an average size of 500 bp using a BioRuptor (Diagenode, Liege, Belgium). After preclearing with a 50% slurry of protein G agarose preincubated with salmon sperm DNA and bovine serum albumin for 4 h at 4°C, the chromatins were incubated withanti-HIC1 antibody (Cat#: H8539, Sigma, St Louis, MO, USA), normal rabbit IgG or with anti-RNA polymeraseⅡ antibody overnight. The antibody bound chromatin was then pulled down for 60 min with protein G agarose, washed extensively, and eluted two times with elution buffer. After addition of 20 μl of 5 M NaCl, the cross-linking was reversed by overnight incubation at 65°C. The immunoprecipitated DNAs as well as whole cell extract DNAs (input) were preliminarily purified by treatment with RNase A and then proteinase K followed by further purification. The purified DNA was used for PCR analyses using the relevant primers for IL-6 and GAPDH. The PCR specific primers for amplification of the IL-6 promoter region at -181/+70containing HIC1-responsive elements (HiREs) as follows: sense primer: 5'-CAATGACGACCTAAGCTGCACT-3'and antisense primer: 5'-CTCTTTCGTTCCCGGTGGGCTC -3'. The PCR products for IL-6 were amplified successfully under the following conditions: initial denaturation, 94°C for 3 min; denaturation, 94°C for 20 sec; annealing, 59°C for 30 sec, extension, 72°C for 30 sec, altogether 32 cycles; 72°C extension for 2 min, and 4°C forever. Human GAPDH gene is used as the reference gene for monitoring the experiment. The following primers were used for GAPDH analysis: sense primer: 5'-TACTAGCGGTTTTACGGGCG-3' and antisense primer: 5'-TCGAACAGGAGGAGCAGAGAGCGA-3'.

References

1. McNabb DS, Reed R and Marciniak RA. Dual luciferase assay system for rapid assessment of gene expression in Saccharomyces cerevisiae. Eukaryotic cell. 2005; 4(9):1539-1549.
2. Liu LJ, Leung KH, Chan DS, Wang YT, Ma DL and Leung CH. Identification of a natural product-like STAT3 dimerization inhibitor by structure-based virtual screening. Cell death & disease. 2014; 5:e1293.

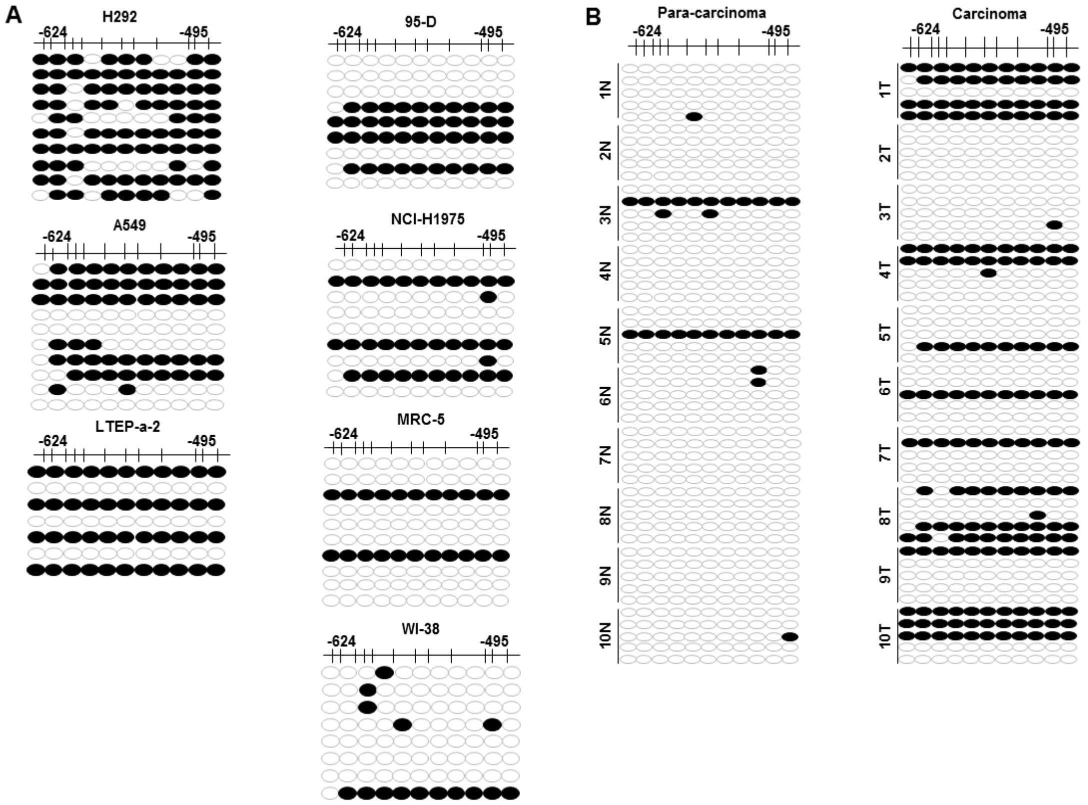

Supplementary Figure 1. The methylation status of 11 CpG sites located in -624 to -495bp of HIC1 promoter in NSCLC cells and tissues were detected by BSP assays.

(A) The status of HIC1 methylation in H292, 95-D, A549, NCI-H1975, LTEP-a-2, MRC-5 and WI-38 cells were detected by BSP assays.

(B) The status of HIC1 methylation in NSCLC and the corresponding para-carcinoma tissues. Black solid circles represented methylation sites; hollow circle represents unmethylation sites.

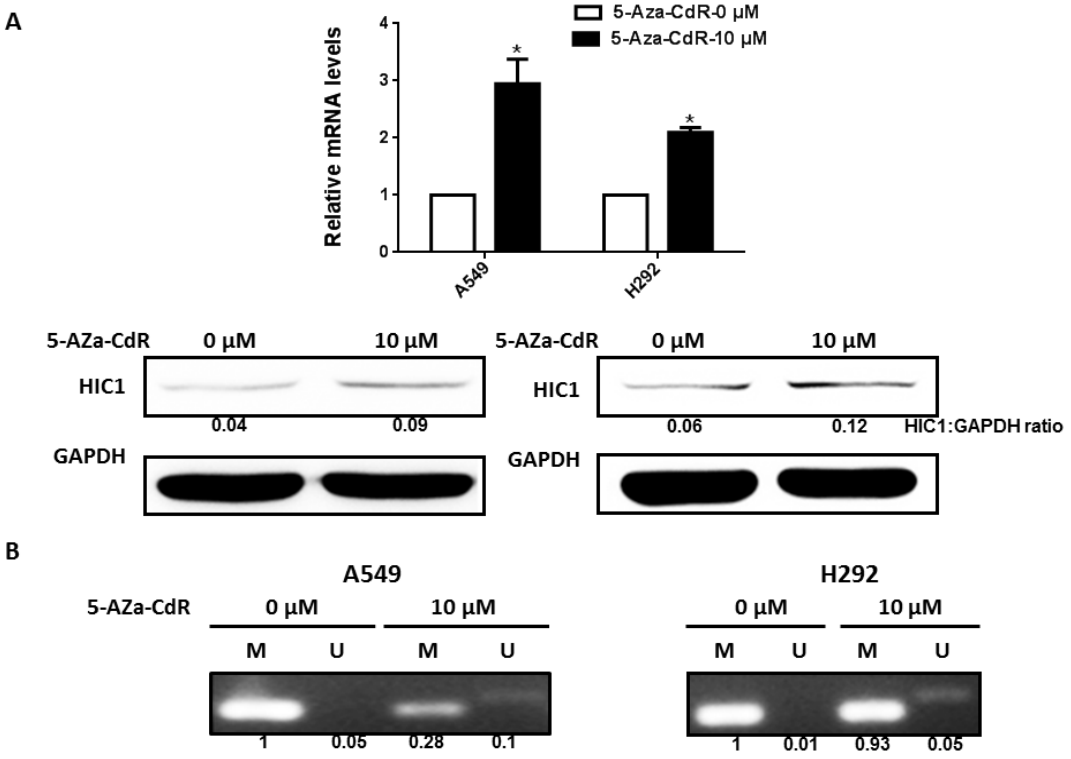

Supplementary Figure 2. Expression of HIC1 is elevated in A549 and H292 cells after treatment with DNMT inhibitor 5-Aza-CdR.

(A) The expressions of HIC1 in A549 and H292 cells treated with or without 10 μM 5-Aza-CdR for 48 h were determined by real-time PCR assay and western blot analysis.

(B) The methylation status of HIC1 promoter in A549 and H292 cells treated with or without 10 μM 5-Aza-CdR for 48 h was determined by MSP assay. M: methylation; U: unmethylation.

Three independent experiments were performed. Data are expressed as mean ± SD. *$p < 0.05$. The intensity of the bands was quantified using software Image-Pro Plus 6.0.

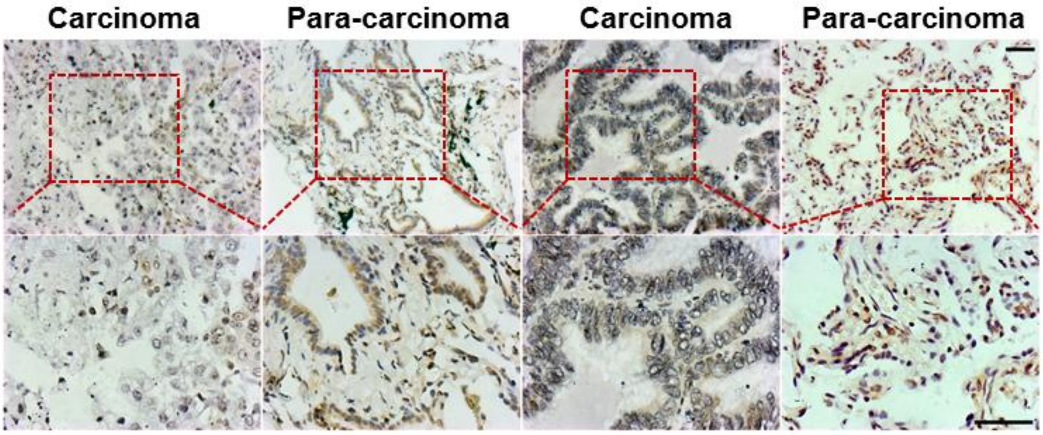

	HIC1 expression			
	Total Cases	Negetive (N%) (0≤score<6)	Positive (N%) (6≤score≤12)	P-Value
**Pathology**				**<0.001**
Para-carcinoma	69	33 (47.8%)	36 (52.2%)	
Carcinoma	65	55 (84.6%)	10 (15.4%)	
**Grade**				**0.011**
1	8	4 (7.4%)	4 (40.0%)	
2	36	31 (57.4%)	5 (50.0%)	
3	20	19 (35.2%)	1 (10.0%)	

Supplementary Figure 3. Expression of HIC1 in NSCLC and the corresponding para-carcinoma tissues was detected by immunohistochemical analysis of tissue microarrays (TMAs).

Representative micrographs of the HIC1 IHC staining in NSCLC and the corresponding para-carcinoma tissues(upper×200, scale bar, 50μm; below×400, scale bar, 50μm).Statistical analysis of HIC1 IHC staining is shown in the below table. The positive expression ratio of nuclear HIC1 was significantly higher in para-carcinoma than in carcinoma (52.2% vs 15.4%, p<0.001). In addition, there was a significant correlation between poorer differentiation grade and nuclear HIC1 expression (p= 0.011).The p value was calculated by chi-square test.

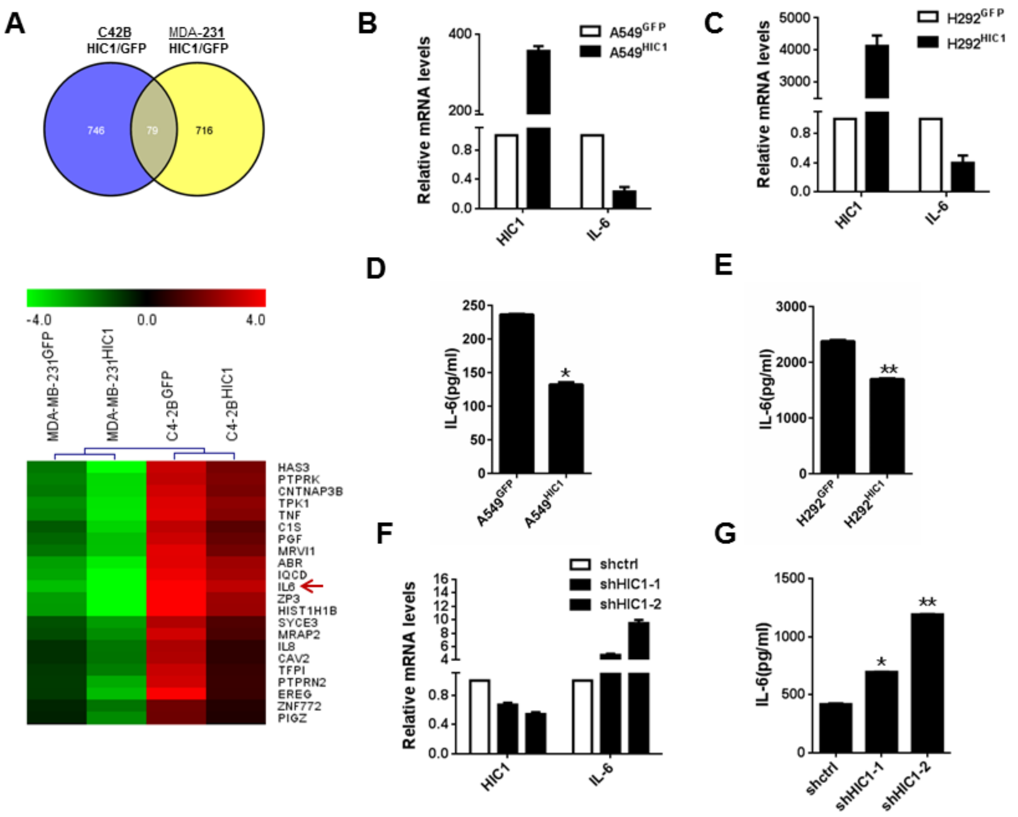

Supplementary Figure 4. HIC1 inhibits the expression of IL-6.

(A) Venn diagram shows the overlapped differentially expressed genes between two cell groups. Fold changes of HIC1/GFP≥2.Clustering heat map shows partial differentially expressed genes. Row represents gene; column represents experimental cells. The red arrow indicates IL-6 gene.

(B) and **(C)**The mRNA expression of HIC1 and IL-6 were both detected by quantitative real-time PCR assays when restoring HIC1 in A549 and H292 cells. The transcription of IL-6 was reduced obviously in both cells.

(D)and **(E)** ELISAs confirm that the protein expression of IL-6 were greatly down-regulated by restoring HIC1 in A549 and H292 cells.

(F) Real-time PCR assay show that IL-6 expression was greatly upregulated in A549 cells by shRNAs-mediated silencing of HIC1.

(G) ELISAs show that IL-6 expression was greatly upregulated in A549 cells by shRNAs-mediated silencing of HIC1.

Three independent experiments were performed. Data are expressed as mean ± SD.*p< 0.05, **p< 0.01.

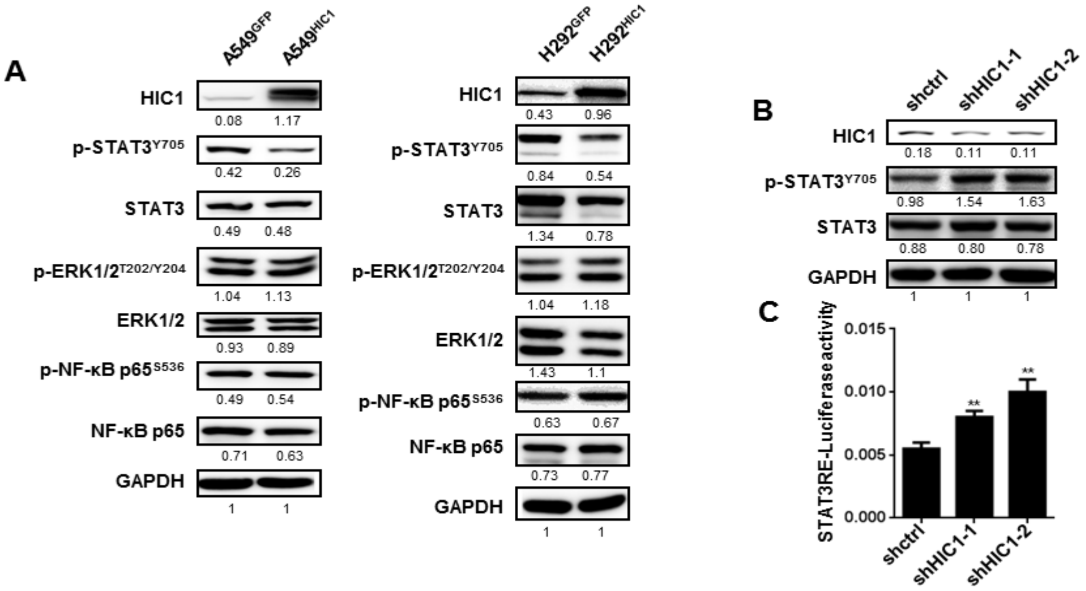

Supplementary Figure 5. HIC1 inhibits the phosphorylation and activity of STAT3.

(A) Expression of p-ERK1/2$^{T202/Y204}$, p-STAT3^{Y705} and p-NF-κB p65^{S536} were detected by western blot analyses in A549^{HIC1}, H292^{HIC1} and the respective control cells.

(B) Expression of p-STAT3^{Y705} and STAT3 were detected by western blot analysis in HIC1 knockdown and control A549 cells, GAPDH was used as a protein loading control.

(C) The activity of STAT3 in HIC1 knockdown and control A549 cells were analyzed by STAT3 response element luciferase reporter assay.

The intensity of the bands was quantified using software Image-Pro Plus 6.0, ratio to GAPDH. Three independent experiments were performed. Data are expressed as mean ± SD. ***p*< 0.01

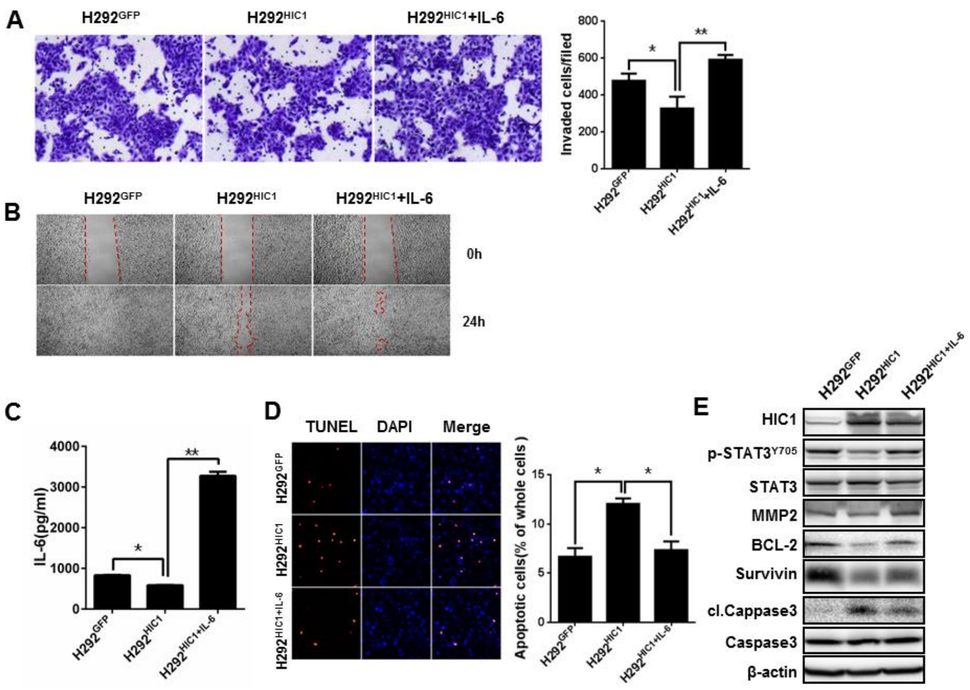

Supplementary Figure 6. IL-6 partially rescues HIC1-induced phenotypes of H292 cells.

(A) Matrigel invasion assay detected the invasion activity of the indicated cells stimulated with or without exogenous IL-6 (40 ng/ml). The average number of cells invaded per field was calculated and displayed on the right histogram.

(B) Scratch healing assay detected the migration activity of the indicated cells stimulated with or without exogenous IL-6 (40 ng/ml).

(C) ELISAs determined the expression of IL-6 in $H292^{GFP}$, $H292^{HIC1}$ and $H292^{HIC1+IL\text{-}6}$ cells.

(D) Apoptosis of $H292^{GFP}$, $H292^{HIC1}$and $H292^{HIC1+IL\text{-}6}$ cells were detected by TUNEL assay. TUNEL positive nuclear is noted as the apoptotic cells; nuclear is counter-stained with DAPI. The relative number of apoptotic cells was displayed on the right histogram.

(E) P-$STAT3^{Y705}$, MMP2, Bcl-2 and Survivin levels were detected in $A549^{GFP}$, $H292^{HIC1}$and $H292^{HIC1+IL\text{-}6}$ cells by western blot analysis.β-actin was used as a protein loading control.

Three independent experiments were performed. Data are expressed as mean ± SD. *p 0.05, **p< 0.01.

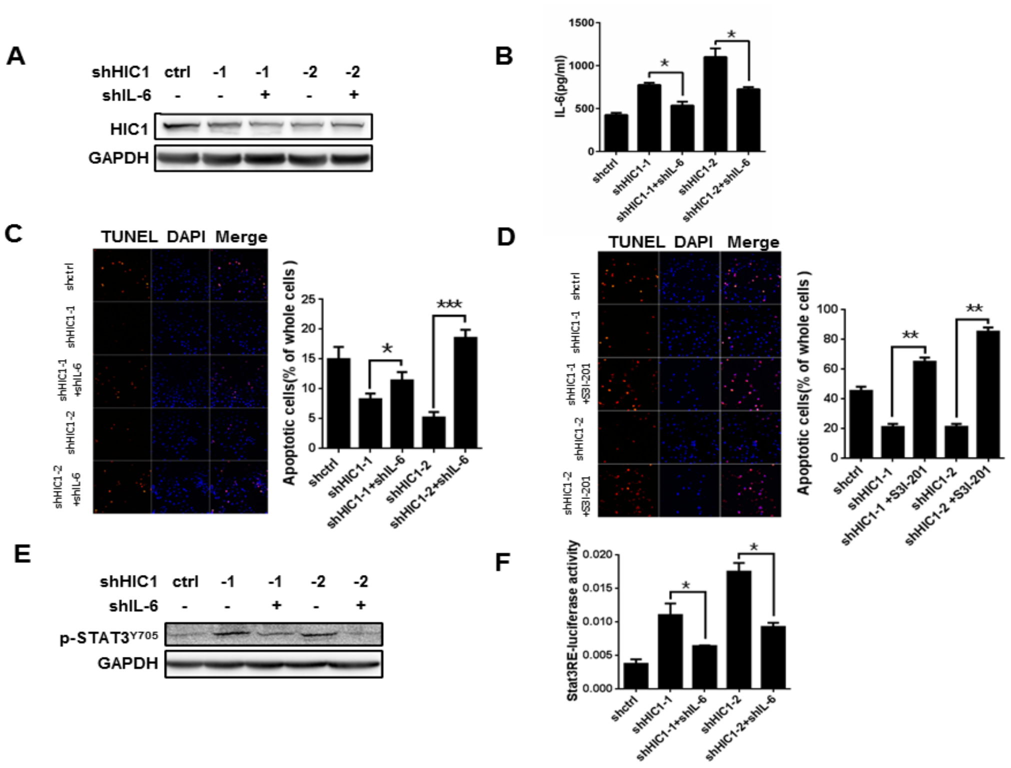

Supplementary Figure 7. Increased expression of IL-6 in A549$^{shHIC1-1/2}$ cells enhances apoptosis through activating STAT3.

(A) Western blot analysis of HIC1 expression in A549 cells stably infected with shctrl, shHIC1-1, shHIC1-1+shIL-6, shHIC1-2 and shHIC-2+shIL-6 vectors. **(B)** ELISAs showed IL-6 level in A549 cells stably infected with shctrl, shHIC1-1, shHIC1-1+shIL-6, shHIC1-2 and shHIC-2+shIL-6 vectors. **(C) and (D)** Tunel assay detected that reduced apoptosis was greatly enhanced in A549$^{shHIC1-1/2+shIL-6}$ cells and A549$^{shHIC1-1/2}$ cells treated with STAT3 inhibitor S3I-201 compared with A549$^{shHIC1-1/2}$ cells. Representative images were shown (× 100). **(E) and (F)** Western blot and luciferase reporter assay showed that the phosphorylation levels and activity of STAT3 were markedly reduced in A549$^{shHIC1-1/2+shIL-6}$ cells compared with the A549$^{shHIC1-1/2}$ cells. Three independent experiments were performed. Data are expressed as mean ± SD* $p < 0.05$, ** $p < 0.01$, *** $p < 0.001$

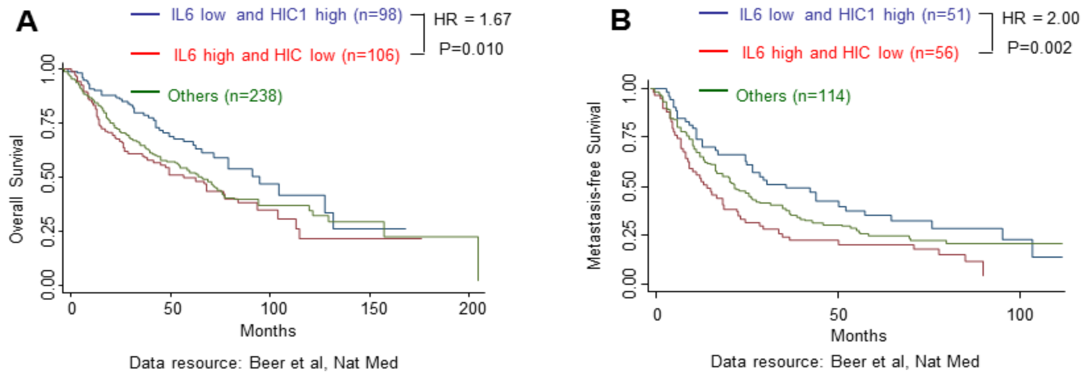

Supplementary Figure 8. HIC1/IL-6 axis predicts overall survival (OS) and metastasis-free survival of NSCLC patients

(A) Kaplan–Meier plots of overall survival (OS) in NSLCC patients, stratified by combined expression of HIC1 and IL-6 in each patient. Data obtained from the clinical microarray database of NSCLC [1]. **(B)** Kaplan–Meier plots of metastasis free survival (MFS) in NSLCC patients, stratified by combined expression of HIC1 and IL-6 in each patient. Data obtained from the clinical microarray database of NSCLC[1].

The p value was calculated by a log rank test. Univariable analyses were conducted, independent of smoking, age, gender, stage, histology.

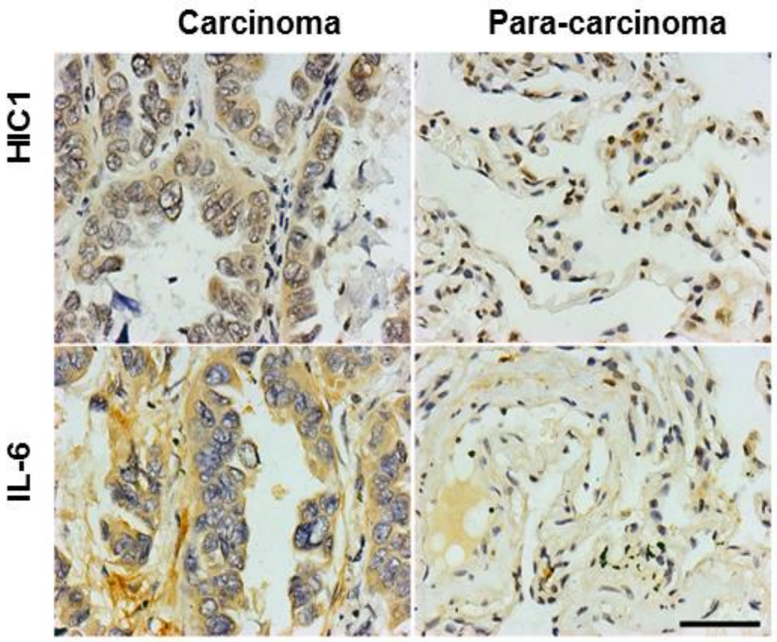

Correlations

			IL-6	HIC1
Spearman's rho	IL-6	Correlation Coefficient	1.000	-.202*
		Sig. (2-tailed)	.	.021
		N	130	130
	HIC1	Correlation Coefficient	-.202*	1.000
		Sig. (2-tailed)	.021	.
		N	130	130

*. Correlation is significant at the 0.05 level (2-tailed).

Supplementary Figure 9. Expression of HIC1 negatively correlates with IL-6 level analyzed by IHC staining of high-density tissue microarray.

IHC analysis showed that the nuclear HIC1 expression level in para-carcinoma was higher than in carcinoma tissues; while the cytoplasmic IL-6 expression level in para-carcinoma was lower than in carcinoma tissues. Representative microscopic images of carcinoma and para-carcinoma tissues stained with an anti-human HIC1 antibody (1:200) and IL-6 (1:200). (×400, scale bar, 50μm). The below table shows the expression level of HIC1 is negatively correlated with IL-6 level using Spearman's correlation analysis in 130 cases of tissues. Values of p=0.021, was considered statistically significant. The correlation coefficient r=-0.202, indicates negative correlation.

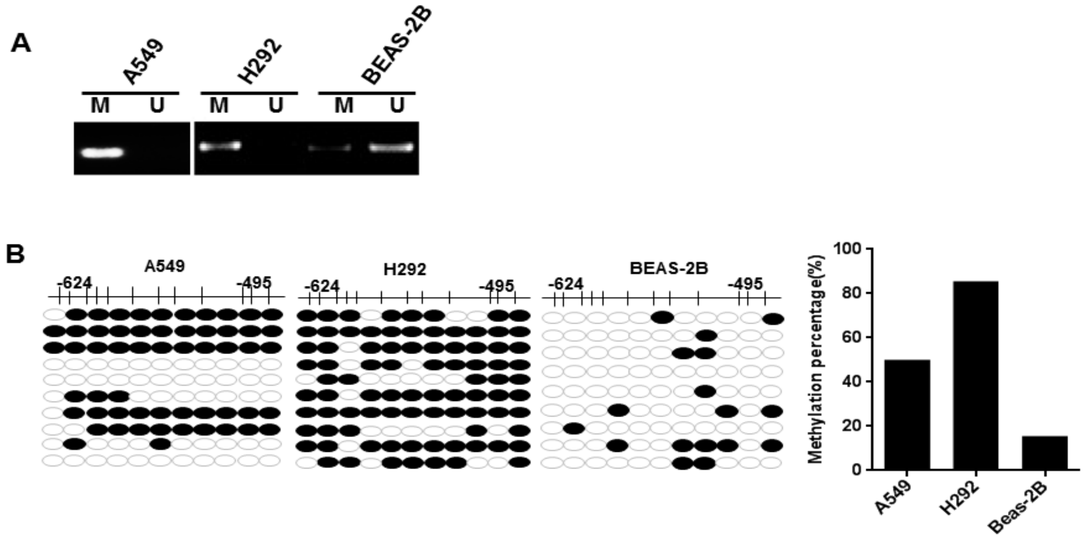

Supplementray Figure 10.The methylation status of HIC1 promoter in A549, H292 and BEAS-2B cells

(A) The status of HIC1 methylation in A549, H292 and BEAS-2B cells were detected by MSP assays. M: methylation; U: unmethylation.

(B) 11 CpG sites located in -624 to -495bp of HIC1 promoter were detected by BSP assays. Black solid circles represented methylation sites; hollow circle represents unmethylation sites. Percentage of methylation was calculated as shown in the histogram.

Supplementary table 1: Quantitative real-time PCR primers

gene	Sense primer	Antisense primer
HIC1	5'-GTCGTGCGACAAGAGCTACAA-3'	5'-CGTTGCTGTGCGAACTTGC-3'
IL-6	5’- CTTCGGTCCAGTTGCCTTCT-3’	5’-AGGAACTCCTTAAAGCTGCG-3’
IL-11	5’-CACAACCTGGATTCCCTG-3’	5’-GCAGGTAGGACAGTAGGT-3’
CNTF	5’-TGACTGCTCTTACGGAATC-3’	5’-GCTTGAAGGTTCTCTTGGA-3’
LIF	5’-CCAACGTGACGGACTTC-3’	5’-TTGCTGTGGAGGCTGAG-3'
IL-6R	5’-GAGGGAGACAGCTCTTTCTAC-3’	5’-CCGTTCAGCCCGATATCTGAG-3’
Gp130	5’-ACCTATGAAGATAGACCATCTAAA-3’	5’-GGTTCTATAAAATATAGTATAATT-3’
GAPDH	5'-ACGGATTTGGTCGTATTGGG-3'	5'-CGCTCCTGGAAGATGGTGAT-3'

Supplementary table 2: Pathological information of lung cancer specimens

NO.	Gender	Age	pathologic types	Grade	TNM staging	Tumor size(cm)
**1**	M	63	SCC	II	T3N2	10*8*6
**2**	M	79	AC	II	T2N0	4*3*2
**3**	M	74	SCC	II	T1N0M0	1*1*0.8
**4**	F	62	AC	II	T4N2M0	3*2*2
**5**	M	79	SCC	II	T1N2	3.5*2*2
**6**	M	63	AC	II	T1N2	2*2*2
**7**	M	62	AC	II	T3N0M0	9*7*3.5
**8**	F	54	AC	II	T1N2M1	2*1*0.5
**9**	M	60	AC	II	T2N2M0	3*3*2
**10**	M	73	AC	II	T3N2M0	7*5*5

M: male F: female

SCC: Lung squamous cell carcinomas AC: Lung adenocarcinoma

References

1. Director's Challenge Consortium for the Molecular Classification of Lung A, Shedden K, Taylor JM, Enkemann SA, Tsao MS, Yeatman TJ, Gerald WL, Eschrich S, Jurisica I, Giordano TJ, Misek DE, Chang AC, Zhu CQ, Strumpf D, Hanash S, Shepherd FA, et al. Gene expression-based survival prediction in lung adenocarcinoma: a multi-site, blinded validation study. Nature medicine. 2008; 14(8):822-827.
